# HIV‐1 testing in the context of expanding PrEP modalities

**DOI:** 10.1002/jia2.26491

**Published:** 2025-07-02

**Authors:** Urvi M. Parikh, Jana L. Jacobs, Njambi Njuguna, Kristine Torjesen, John W. Mellors

**Affiliations:** ^1^ Division of Infectious Diseases Department of Medicine University of Pittsburgh Pittsburgh Pennsylvania USA; ^2^ FHI 360 Kenya Nairobi Kenya; ^3^ FHI 360 Durham North Carolina USA

**Keywords:** HIV prevention, HIV‐1 diagnostics, HIV testing algorithms, long‐acting cabotegravir, pre‐exposure prophylaxis, rapid diagnostic tests

## Abstract

**Introduction:**

Multiple effective antiretroviral‐based pre‐exposure prophylaxis (PrEP) modalities for HIV‐1 prevention are now available or under investigation but their safe rollout requires implementable HIV‐1 testing strategies that accurately identify rare cases of HIV‐1 acquisition. Current PrEP testing guidelines and testing algorithms in PrEP studies are varied, using single or combinations of rapid antibody‐based diagnostic testing, qualitative or quantitative nucleic acid testing, and/or sample collection for retrospective analyses with sensitive research assays for HIV‐1 nucleic acid detection. The objective of this commentary is to summarize current and alternative HIV testing approaches for PrEP implementation to guide best practices for individual programmes.

**Discussion:**

Diagnosing HIV‐1 in PrEP users is challenging because (1) rarity of breakthrough HIV‐1 in individuals on PrEP that increases the risk of a false‐positive test; (2) modification of acute HIV infection by PrEP; and (3) PrEP delivery in community settings with inadequate testing infrastructure. Current best practices indicate the use of rapid diagnostic tests or self‐testing as recommended by national testing algorithms and the World Health Organization (WHO). The use of nucleic acid testing such as plasma HIV‐1 RNA polymerase chain reaction may allow earlier detection of HIV‐1 but feasibility and risk of false positive are downsides. Sensitive tests to detect single‐copy HIV‐1 RNA in plasma and integrated proviral DNA in blood mononuclear cells may be important methods to resolve ambiguous HIV‐1 diagnosis in research settings. Delayed diagnoses could lead to drug resistance emergence under long‐acting PrEP selection, whereas single unconfirmed false‐positive tests could create diagnostic challenges in users of long‐acting PrEP. The cost, feasibility and positive predictive value of HIV tests are important considerations for PrEP programmes.

**Conclusions:**

Optimal strategies to detect HIV‐1 acquisition among users of different PrEP modalities are evolving. While new guidance from the WHO recommends HIV‐1 testing by serological assays or self‐testing with PrEP use, feasible plans for clinical management of rare cases of breakthrough on PrEP and ambiguous diagnoses are still needed. The data from PrEP studies and scale‐up will help us assess the value of different tests and testing approaches for their inclusion in HIV detection algorithms across PrEP modalities.

## 1 INTRODUCTION

### 1.1 The need for global scale‐up of pre‐exposure prophylaxis modalities

Multiple pre‐exposure prophylaxis (PrEP) modalities are currently approved for the prevention of HIV‐1 acquisition among individuals at risk, including daily oral tenofovir disoproxil fumarate (TDF) or tenofovir alafenamide (TAF) with emtricitabine (FTC) or lamivudine (3TC), monthly dapivirine intravaginal rings and long‐acting injectable cabotegravir (CAB PrEP) for HIV‐1 prevention [1]. Lenacapavir is under investigation as a 6‐monthly injectable for PrEP, with no acquisitions observed among cisgender adolescent girls and young women in the twice‐yearly lenacapavir arm of the PURPOSE 1 study in South Africa and Uganda [2]. Only two incident HIV‐1 acquisitions were observed among 2179 participants in the twice‐yearly lenacapavir arm of PURPOSE 2, which included cisgender men and gender‐diverse persons [3].

In addition, promising agents in development include a 3‐monthly dapivirine ring, multi‐purpose technologies that include hormonal contraception or drugs for sexually transmitted infection prevention, and new PrEP modalities such as implants and films [4, 5, 6, 7, 8, 9, 10]. PrEP is highly efficacious at preventing HIV‐1 acquisition, with effectiveness ranging with modality and product use. Barriers to expanded use and scale‐up include the burden and costs of healthcare provider visits and the intensive requirement of HIV‐1 testing before PrEP initiation and frequent re‐testing during PrEP continuation [11, 12].

### 1.2 Varied HIV‐1 testing approaches for PrEP in the United States and globally

Current United States guidelines recommend a plasma antibody/antigen laboratory‐based test or a fingerstick‐drawn antigen/antibody rapid diagnostic test (RDT) prior to CAB PrEP initiation for individuals who have not taken oral PrEP or post‐exposure prophylaxis (PEP) in the last 3 months, or who have not received a CAB PrEP injection in the last 12 months. By contrast, individuals switching from oral PrEP or PEP to CAB PrEP, or individuals continuing or restarting CAB PrEP after a gap of up to 12 months, are recommended to have both an HIV‐1 antibody/antigen test and a qualitative or quantitative HIV‐1 ribonucleic acid (RNA) assay with each CAB injection [13]. The option of using RDT or laboratory‐based tests allows HIV testing in both clinic and community‐based settings (summarized in Table 1).

**Table 1:** Parameters for pre‐exposure prophylaxis (PrEP) administration, testing and overlap with antiretroviral therapy by PrEP modality

Parameter	TDF/FTC OR TAF/FTC	Dapivirine ring^a^	Cabotegravir	Lenacapavir^b^
Drug class	NRTI	NNRTI	INSTI	Capsid inhibitor
Overlap with inhibitors used for treatment	TDF/FTC and a third ARV (e.g. efavirenz, elvitegravir or dolutegravir) TDF with two other ARVs TAF/FTC with 1–2 other ARVs	None	CAB/rilpivirine	Lenacapavir
Frequency of administration	Daily oral dosing Oral event‐driven PrEP^c^	Once monthly vaginal ring	Initiation with single 600 mg/3 ml intramuscular injection Second injection 1 month ± 7 days later Subsequent injections every 2 months ± 7 days	Twice yearly
Suggested HIV testing frequency	Every 3 months (CDC) One month after PrEP initiation then every 3 months (WHO)	Every 3 months	Every 2 months, with each injection	No guidance yet
HIV testing algorithm	Follow local standard‐of‐care	Follow local standard‐of‐care	Ab/Ag test for initiation; Ag/Ag test AND RNA test for follow‐up (United States only) Follow local standard‐of‐care (WHO)	No guidance yet

Abbreviations: Ag/Ab, antigen/antibody; ARV, antiretroviral; CAB, cabotegravir; CDC, United States Centers for Disease Control and Prevention; INSTI, integrase strand transfer inhibitor; NNRTI, non‐nucleoside reverse transcriptase inhibitor; NRTI, nucleoside/nucleotide reverse transcriptase inhibitor; PrEP, pre‐exposure prophylaxis; RNA, ribonucleic acid; TAF/FTC, tenofovir alafenamide/emtricitabine; TDF/FTC, tenofovir disoproxil fumarate/emtricitabine; WHO, World Health Organization.

^a^ European Medicines Agency has provided a positive benefit‐risk opinion on dapivirine ring. The World Health Organization (WHO) has recommended dapivirine ring for HIV prevention; dapivirine ring is not approved by the U.S. Food and Drug Administration.

^b^ Under investigation; no regulatory bodies have approved as of March 2025.

^c^ Event‐driven dosing is off‐label in the United States. Guidance for event‐driven dosing is provided by WHO [14].

The World Health Organization (WHO) recommends conducting testing according to each country's national testing algorithm for all forms of PrEP and does not recommend the use of quantitative HIV‐1 RNA tests prior to PrEP initiation or continuation [15]. Recently, WHO included self‐testing as an option for starting, re‐starting or continuing PrEP, to expand access, reduce barriers to PrEP and simplify service delivery [16].

Implementation studies that include CAB as a PrEP option have had diverse approaches to HIV‐1 testing due to study design or resources available, such as requiring undetectable HIV‐1 RNA prior to CAB injection using a same‐day assay such as Gene Xpert or collecting blood for future retrospective HIV‐1 RNA testing [17, 18, 19]. Evaluation of stored samples from studies will contribute to the generation of data for determining optimal approaches.

### 1.3 Testing challenges and risk of drug resistance with HIV‐1 acquired on PrEP modalities

Despite the high efficacy of PrEP when used as prescribed, breakthrough HIV‐1 acquisitions have occurred rarely on each of the different PrEP modalities even with evidence of adherence [20, 21]. The challenge of diagnosing HIV‐1 on PrEP and the relative risk of HIV‐1 resistance to the PrEP agent differ by PrEP modality, which adds further complexity.

For dapivirine ring, the risk of drug resistance in two Phase III studies did not differ between participants who acquired HIV‐1 on the active or placebo study arms, and the rate of resistance among those who seroconverted during open‐label dapivirine ring studies was similar to the background prevalence of pre‐treatment non‐nucleoside reverse transcriptase inhibitor (NNRTI) drug resistance at the time the studies were conducted [20, 22–24]. Dapivirine concentrates in vaginal tissue during ring use but achieves low systemic levels, which together may reduce the risk of systemic resistance if the ring is inadvertently started during undetected acute HIV‐1 infection, compared to the resistance risk with oral PrEP or CAB PrEP if started during undetected acute infection [25, 26]. Furthermore, the current globally recommended first‐line antiretroviral therapy (ART) regimen of tenofovir, lamivudine and dolutegravir (TLD) does not have overlapping resistance profiles with dapivirine, indicating that TLD should be effective for treatment in individuals with NNRTI‐resistant HIV‐1 [27] (Table 1).

In the decade since TDF/FTC was approved as oral PrEP, PrEP‐associated resistance (i.e. mutations K65R, M184I and/or M184V in HIV‐1 *reverse transcriptase*) were observed in 61 of 310 reported seroconversions from 10 implementation projects and/or programmatic rollout settings globally [28]. Clinical studies have demonstrated a higher risk of HIV‐1 resistance when oral TDF/FTC PrEP is started during undiagnosed acute infection [20, 29]. The Partners PrEP study noted a significant increase in delayed detection of HIV‐1 acquisition up to 1 month in ongoing oral PrEP users; however, this delay was not associated with a higher risk of resistance [30].

Six individuals from SeroPrEP are reported to have seroconverted on CAB PrEP despite on‐time injections in HPTN 083, and all developed combinations of major integrase strand transfer inhibitor (INSTI) resistance mutations [31, 32, 33]. SeroPrEP is an ongoing study of HIV‐1 acquisition on PrEP and subsequent treatment outcomes in routine care in the United States. In six individuals who acquired HIV‐1 despite on‐time CAB injections, three who started ART within 2 weeks of diagnosis had low‐frequency HIV‐1 INSTI‐associated mutations, while one individual who had delayed ART start by 56 days had multiple major HIV‐1 INSTI‐associated mutations, suggesting a delayed diagnosis or delayed switch to ART after acquiring HIV‐1 could increase the risk of INSTI resistance [34, 35, 36].

Several pathways of resistance have been observed from treatment trials of lenacapavir [37]. Nine participants with emergent lenacapavir resistance by week 52 in the Phase 2/3 CAPELLA study evaluating the efficacy and safety of lenacapavir‐based therapy in individuals with multi‐drug resistance HIV‐1, had combinations of one to four capsid‐associated mutations including M66I, Q67H, K70H/N/R/S, N74D, A105T and/or T107C. M66I occurred most commonly, and conferred a 234‐fold decrease in lenacapavir susceptibility as determined by phenotyping analysis [38, 39]. In the Phase 2 CALIBRATE study of treatment‐naïve individuals receiving lenacapavir‐containing therapy, two individuals with emergent resistance had Q67H alone or with K70R [40]. The mechanisms for PrEP failure and resistance selection with the use of injectable lenacapavir for HIV prevention are not yet known. In the PURPOSE 2 HIV prevention study, only two individuals seroconverted on injectable lenacapavir, and both had HIV‐1 with the N74D capsid resistance‐associated mutation. Mutations selected in treatment‐naïve individuals starting lenacapavir‐containing combination therapy, or in therapy‐experienced individuals switching to lenacapavir combination therapy may not be predictably extrapolated to provide insight on resistance risk for individuals with breakthrough HIV acquisition on lenacapavir PrEP. As the use of capsid inhibitors for both treatment and prevention expands, monitoring for capsid resistance could emerge as a future need to preserve the efficacy of this class of antiretroviral agents.

Injectable PrEP has brought further attention to the challenge of delayed detection of HIV‐1 acquisition, with detection of HIV‐1 acquisition delayed up to 4 months as evidenced by retrospective testing of HIV‐1 RNA from stored plasma by sensitive methods [31, 32, 33]. The HPTN 083 investigators described PrEP‐modified acute infection as having “smouldering” viral replication, diminished or delayed antibody production, and potential for reversion of positive test results in diagnostic assays, with weeks to months before HIV‐1 is detected [41]. The two acquisitions of HIV‐1 that occurred in the 6‐monthly injectable lenacapavir arm of PURPOSE 2 were both detected by routine diagnostic testing; further research is needed to determine if PrEP‐modified acute infection will occur in individuals who acquire HIV‐1 on lenacapavir [3]. Although the risk of pre‐treatment capsid inhibitor resistance is currently low, continued drug resistance monitoring in research settings is essential with the scale‐up of capsid inhibitors for both treatment and prevention.

### 1.4 HIV testing guidelines are needed across PrEP modalities

Optimal approaches to HIV‐1 testing and HIV‐1 detection for different PrEP modalities are not well defined, particularly with long‐acting injectable agents due to the possibility of PrEP‐modified acute HIV‐1 infection. We provide below an overview of the currently available test options to detect HIV‐1 in PrEP users with consideration to feasibility and discuss additional approaches for HIV‐1 diagnosis. We highlight that more data is needed to develop evidence‐based HIV‐1 testing guidance for current and future PrEP modalities, particularly for cases of ambiguous HIV‐1 diagnosis.

## 2 DISCUSSION

### 2.1 Advantages and disadvantages of currently available HIV‐1 tests to detect acute HIV‐1 in individuals using PrEP

The WHO recommends a three‐test strategy for HIV diagnosis using any combination of available RDT or laboratory‐based tests [16]. RDTs conducted using a fingerstick reduce healthcare burden and are more amenable to community settings for PrEP delivery compared to tests using blood draws that require trained phlebotomists [42]. The false‐negative window period can vary among assays; the median window period between HIV exposure and immunoassay reactivity for IgG or IgG/IgM‐based diagnostic tests ranges from 19 to 35 days, which may be extended by weeks to months when used to detect HIV‐1 in users of long‐acting injectable PrEP as demonstrated in HPTN 083 [41, 43, 44]. Furthermore, retrospective analysis of PrEP and early ART initiation studies revealed that exposure to antiretrovirals pre‐seroconversion can reduce or delay HIV‐1 detectability [45]. Similar to other diagnostic tests, the frequency of false‐positive RDTs may also increase as HIV‐1 incidence is reduced in the population of PrEP users [46].

The false‐negative window period for HIV‐1 detection with nucleic acid testing such as HIV‐1 RNA polymerase chain reaction is shorter (10−33 days post‐acquisition) than it is for RDTs, which may prevent some inadvertent PrEP starts during acute HIV‐1 infection [47, 48]. However, the use of HIV‐1 RNA monitoring for PrEP initiation and/or continuation poses significant challenges. In both low/middle‐income and high‐income countries, HIV‐1 RNA testing is performed in central labs. It requires blood collection by venipuncture and adequate infrastructure to process samples to plasma and then ship them frozen or at 4^°^C, or to transport whole blood at ambient temperature within 24 hours to a testing facility [16]. The turnaround time of days to weeks for the return of results can significantly restrict PrEP initiation, potentially resulting in HIV‐1 acquisitions that could have been prevented with an immediate PrEP start.

Capacity for HIV‐1 RNA testing remains suboptimal in low/middle‐income settings as countries strive to reach the UNAIDS 95‐95‐95 treatment goals; HIV‐1 RNA testing is prioritized for individuals on ART and may not be accessible for PrEP users except through a study [49]. In high‐income countries where HIV‐1 RNA tests are routinely available, cost and insurance coverage remain barriers [50]. Most important, quantitative HIV‐1 RNA testing is not licensed for HIV‐1 diagnosis; thus, a single detectable HIV‐1 RNA result cannot replace the need to fulfil a diagnostic algorithm because false‐positive HIV‐1 RNA results do occur. Modelling studies did not predict an advantage of using nucleic acid tests (NAT) over standard antibody‐based testing [51]. A retrospective analysis of the HPTN 083 open‐label extension study found that the positive predictive value of RNA in detecting HIV‐1 acquisition was reduced in individuals exposed to CAB [52]. A national cohort study in the United States of over 30,000 oral and CAB PrEP users reported a false‐positive rate of 0.04% for HIV‐1 RNA tests [53]. Thus, the cost, feasibility and accuracy of HIV‐1 RNA testing in PrEP users must be weighed against its potential to detect HIV‐1 earlier when considering its inclusion in HIV‐1 testing algorithms for current and future long‐acting PrEP.

Research tests with a higher sensitivity to detect the low levels of HIV‐1 characteristic of early HIV‐1 acquisition on PrEP could play an important role in characterizing PrEP‐modified acute HIV‐1 infection, particularly when stored samples from clinical and implementation studies are available to test retrospectively. For example, tests that can detect a single copy of HIV‐1 RNA in plasma could identify HIV‐1 weeks to months earlier than commercially available tests, as demonstrated in HPTN 083 [31, 54, 55]. Similarly, tests that can detect three or more copies of integrated HIV‐1 deoxyribonucleic acid (DNA) in one million blood mononuclear cells could provide evidence of HIV‐1 acquisition even in the absence of detectable plasma HIV‐1 RNA [56, 57, 58]. Conversely, the combination of no detectable HIV‐1 RNA and HIV‐1 DNA by very sensitive assays (1–3 copies) could increase the likelihood that an HIV‐1‐negative result is accurate. Currently, these sensitive and specialized research assays are being used to detect and characterize HIV‐1 acquisition when commercial assay results are ambiguous, but such research assays cannot yet be incorporated into diagnostic algorithms or be used for patient care.

### 2.2 Diagnostic dilemmas and potential solutions

As the use of long‐acting PrEP expands, cases of ambiguous diagnosis are expected to increase. Different iterations of discordant or discrepant HIV‐1 test results are likely to occur as CAB PrEP is rolled out globally although they should remain relatively infrequent overall [34, 53, 59].

No current data is available to inform the best possible testing approaches for cases of unconfirmed HIV‐1 diagnosis, for example a single positive test result (antibody or RNA) with no repeat positive results. With the current lack of consistent guidance combined with suboptimal HIV‐1 testing options, PrEP implementation studies and programmes may consider the following approaches when routine diagnostic algorithms provide equivocal findings (due to delayed seroconversion or lack of sensitivity). (1) Clients could opt to remain on PrEP. If HIV‐1 RNA testing is done, repeat detectable RNA results from independently collected samples may be needed to rule out false‐positive results. (2) Clients could opt to discontinue PrEP and re‐test in 2 or more weeks. They could be at risk of HIV acquisition if no longer taking PrEP, and it would not be possible to distinguish if a new acquisition had occurred after PrEP discontinuation. (3) Clients could switch to ART. ART may not be available in some settings without a true diagnosis and may preclude the confirmation of HIV acquisition if the use of ART further delays or suppresses seroconversion. (4) If available, research tests not currently approved for diagnosis, such as more sensitive HIV‐1 RNA or DNA detection, may be considered in the context of research studies. All research testing should be followed by continued testing for eventual clinical diagnosis (seroconversion or qualitative HIV‐1 nucleic acid detection) after PrEP discontinuation.

The psychological impact of prolonged uncertainty of an unconfirmed HIV‐1 diagnosis and extensive follow‐up testing especially in individuals who expected to be protected from HIV‐1 through their use of PrEP, is not known. Client counselling and provider support remain essential components of PrEP provision, especially in interpreting HIV‐1 test results, understanding the limitations of each test in the context of PrEP, determining how to use results from research‐only tests that are not validated for clinical care but may indicate HIV‐1 acquisition, and helping clients cope with the uncertainty and distress of ambiguous HIV‐1 diagnoses.

### 2.3 Research gaps

Much remains to be learned about optimal HIV‐1 diagnostic approaches with current and future long‐acting PrEP modalities. The role and optimization of self‐testing which has the potential to improve PrEP access, simplify PrEP delivery and reduce clinic burden should be further explored. However, self‐tests may detect HIV‐1 later than facility‐based algorithms, which could prolong diagnosis and increase the risk of drug resistance with long‐acting products [60]. Conversely, quantitative HIV RNA can detect HIV acquisition earlier, but false‐positive HIV test results create a burden on patients and programmes and increase the cost. Sensitive measures to detect HIV‐1 RNA, or integrated HIV‐1 DNA in cells are not validated for clinical diagnosis, and further research is needed. The best methods to resolve HIV ambiguous diagnoses in clinical practice is another important research gap.

## 3 CONCLUSIONS

Antiretrovirals such as dapivirine, TAF/FTC or TDF/FTC, cabotegravir and lenacapavir have tremendous potential as PrEP modalities and are likely to become integral components of global efforts to end the HIV‐1 epidemic. Sensitive, accurate and feasible HIV‐1 testing strategies must be defined and implemented to ensure successful PrEP programmes. PrEP implementation projects and rollout programmes, using current national algorithms to deliver PrEP while collecting samples and data to evaluate rare cases of potential HIV‐1 acquisition, will provide essential data to develop evidence‐based HIV‐1 testing guidance for current and future PrEP modalities.

## COMPETING INTERESTS

UMP is a a consultant to Merck and has previously received a travel honorarium for a conference presentation from Thermo‐Fisher. JWM is a consultant to Gilead Sciences, Inc., and has received grant funding from Gilead Sciences, Inc., to the University of Pittsburgh (unrelated to the current work); receives compensation from Galapagos NV (unrelated to the current work); and holds share options in Galapagos NV, Infectious Disease Connect, Inc., and MingMeg Biotechnology Co., Ltd. (unrelated to the current work). No competing interests are reported by JLJ, NN or KT.

## AUTHORS’ CONTRIBUTIONS

UMP, JWM and JLJ wrote the manuscript; NN contributed to manuscript development; and KT and JWM provided technical input, editing and review of the manuscript.

## FUNDING

This project was made possible by the generous support of the American people through the U.S. Agency for International Development (USAID), in partnership with the President's Emergency Plan for AIDS Relief (PEPFAR), under the terms of Cooperative Agreement numbers AID‐OAA‐A‐15‐00031 and 7200AA21CA00011. The writing of this manuscript was also supported by the National Institute of Allergy and Infectious Diseases of the National Institutes of Health under Award Numbers P30AI036219 and R01AI167753.

## DISCLAIMER

The contents of this paper are the responsibility of the authors and do not necessarily reflect the official views of USAID, PEPFAR, the National Institute of Allergy and Infectious Diseases, the National Institutes of Health or the United States Government.

## Data Availability

Data sharing is not applicable to this article as no datasets were generated or analysed during the current study.
